# Application of Linear Gradient Magnetic Field in Arterial Profile Scanning Imaging

**DOI:** 10.3390/s20164547

**Published:** 2020-08-13

**Authors:** Yanjun Liu, Guoqiang Liu, Dan Yang, Bin Xu

**Affiliations:** 1 College of Information Science and Engineering, Northeastern University, Shenyang 110819, China; 1800720@stu.neu.edu.cn; 2 Institute of Electrical Engineering Chinese Academy of Sciences, Beijing 100190, China; 3 School of Electronics, Electrical and Communication Engineering, University of Chinese Academy of Sciences, Beijing 100190, China; 4 Key Laboratory of Data Analytics and Optimization for Smart Industry MOE, Northeastern University, Shenyang 110819, China; 5 Liaoning Province Key Laboratory of Infrared Optoelectric Materials and Micro-Nano Devices, Northeastern University, Shenyang 110819, China; 6 College of Computer Science and Engineering, Northeastern University, Shenyang 110819, China; xubin@mail.neu.edu.cn

**Keywords:** electromagnetic sensor, linear gradient magnetic field, artery stenosis, finite element, magnetoelectric effects of blood flow, reciprocal theory

## Abstract

*Background and Objectives*: Cardiovascular and cerebrovascular diseases caused by arterial stenosis and sclerosis are the main causes of human death. Although there are mature diagnostic techniques in clinical practice, they are not suitable for early disease prediction and monitoring due to their high cost and complex operation. The purpose of this paper is to study the coupling effect of arterial blood flow and linear gradient magnetic field, and to propose a method for the reconstruction of the arterial profile, which will lay a theoretical foundation for new electromagnetic artery scanning imaging technology. *Methods and Models*: A combination coil composed of gradient coils and drive coils is applied as a magnetic field excitation source. By controlling the excitation current, a linearly gradient magnetic field with a line-shaped zero magnetic field is generated, and the zero magnetic field is driven to scan in a specific direction. According to the magnetoelectric effect of blood flow, under the action of the external magnetic field, the voltage signals on the body surface can be detected by measuring electrodes. The location of the artery center line can be determined by the time–space relationship between voltage signals and zero magnetic field scanning. In addition, based on the reciprocity theorem integral equation, a numerical model between the amplitude of the voltage signal and the arterial radius is derived to reconstruct the arterial radius. The above physical process was simulated in the finite element analysis software COMSOL, and the voltage signals obtained from the simulation verified the arterial profile reconstruction. *Results*: Through finite element simulation verification, the imaging method based on a linear gradient magnetic field has a numerical accuracy of 90% and a spatial resolution of 1 mm. Moreover, under 100 Hz low-frequency alternating current excitation, the single scanning time is 0.005 s, which is far shorter than the arterial blood flow change cycle, meeting the requirements of real-time imaging. The results demonstrate the effectiveness and high theoretical feasibility of the proposed method in real-time arterial imaging. *Conclusions:* This study indicates the potential application of linear gradient magnetic fields in arterial profile imaging. Compared with traditional electromagnetic imaging methods, the proposed method has the advantages of fast imaging speed and high resolution, showing the certain application value in early real-time imaging of arterial disease. However, further studies are necessary to confirm its effectiveness in clinical practice by more medical data and real cases.

## 1. Introduction

According to investigation [1], cardiovascular and cerebrovascular diseases are the main causes of human death, and the main cause of cardiovascular and cerebrovascular diseases such as coronary heart disease and angina is atherosclerosis, which leads to arterial stenosis and affects the normal circulation of blood. At present, the commonly used diagnostic techniques for arterial stenosis include digital subtraction angiography (DSA), CT angiography (CTA), magnetic resonance angiography (MRA), and medical ultrasound examination. DSA is the gold standard of angiography, but because of its high trauma, it is generally used for preoperative examination [2,3]. CTA has the advantages of fast imaging and low trauma, but it still needs to inject a contrast agent, and X-ray has some potential harm to human body [4,5]. MRA is currently considered non-invasive and safe, but its diagnosis is costly and therefore not suitable for routine medical examinations [6,7]. Medical ultrasound examination technology has relatively low cost, high resolution, and can be used for real-time imaging. However, its disadvantages lie in the poor penetration of ultrasound to bone, extremely limited brain imaging, and the operator’s manipulation is very important [8,9]. Superb skills and rich experience are necessary for obtaining high-quality images and making accurate diagnosis. Therefore, it is still expected to develop a non-invasive, low-cost, fast imaging, high-resolution, and easy to operate angiography technology, which can be applied to the early diagnosis of diseases in the future, even using portable, home-based diagnostic equipment.

In recent years, electromagnetic imaging technology for medical diagnosis has made great progress, and many novel imaging technologies have emerged, such as electrical impedance tomography, magnetic acoustic tomography, magneto-acousto-electrical tomography, magnetic induction tomography, thermoacoustic tomography, and so on [10,11,12,13,14,15]. Dr. Ruben Specogna and Dr. Antonio Affanni et al. effectively fused optical and impedance data and proposed novel biosensor and inversion technology for monitoring and predicting thrombosis characteristics, which provided a new idea for electromagnetic arterial blood flow imaging [16,17]. In 2016, Dr. Ali of The University of Huddersfield verified the linear relationship between electrode potential difference signal and blood flow under a uniform magnetic field through numerical simulation and experiment [18], which greatly promoted the research progress of the electromagnetic non-invasive blood flow measurement method.

In addition, 3D reconstruction technology has made significant progress in vascular diagnosis and treatment in recent years. Dr. Andrzej Polanczyk et al. proposed a method to reconstruct the 3D geometry of blood vessels using computational fluid dynamics and medical data to predict the outcome after surgery, and the comparison with real postoperative data shows that the reconstruction method has a high accuracy [19]. In fact, 3D reconstruction technology can not only be applied in clinical diagnosis and treatment, it can also be used as an important tool in the research of cutting-edge medical imaging technology. For example, it could combine digital twinning technology to build more realistic vascular simulation models, which would greatly facilitate non-medical researchers and promote the development of interdisciplinary research between medicine and engineering. It should be noted that this study focuses on the arterial imaging method based on the magnetoelectric effect of blood flow, so the finite element vascular model with electromagnetic parameters and flow velocity parameters is adopted. The influence of more hydrodynamic parameters will be considered in future studies.

In our previous work [20,21], based on the reciprocity theorem, we derived the integral equation of the surface potential signal and arterial blood flow velocity in detail and realized arterial imaging by inverting blood flow velocity distribution. However, this imaging method itself is an underdetermined and ill-conditioned problem. The higher resolution depends on more electrodes to obtain more surface potential data, and a large number of operations are required in the inversion process, resulting in the slow imaging speed, which is also one of the common problems of inversion imaging technology. Therefore, an arterial profile scanning imaging method based on a linear gradient magnetic field is proposed in this paper. A linear gradient magnetic field has been widely used in magnetic particle imaging [22], but the application of arterial flow imaging has not been studied so far. The advantage of this method is that the imaging speed is fast and does not require a lot of inversion calculation. Moreover, there is no need to generate a uniform magnetic field in the measurement area, so the volume of the excitation coil is saved, which is conducive to the miniaturization of the detection system.

The purpose of this paper is to study the coupling effect of arterial blood flow and a linear gradient magnetic field, and to propose a method for reconstruction of the arterial profile, which will lay a theoretical foundation for new electromagnetic artery scanning imaging technology. The organizational structure and main contributions of this paper are as follows. A combined coil structure that can drive rapid scanning of the zero magnetic field region is introduced in Section 2.2, a numerical model between the amplitude of the surface potential signal and the artery radius is derived in Section 2.3, and the algorithm process of arterial profile scanning imaging is introduced in Section 2.4. The finite element simulation results and the final imaging results are analyzed in Section 3. Finally, in Section 4, the general conclusions are summarized, and the future research direction is prospected.

## 2. Methods and Models

### 2.1. Basic Electromagnetic Theory

#### 2.1.1. Magnetoelectric Effects of Blood Flow

Arterial blood flow has both fluidity and electrical conductivity. When the direction of the external magnetic field is perpendicular to the direction of blood flow, according to Faraday’s law, the blood flow velocity will be coupled with the magnetic field to form a current source $J_{e}$:

(1) $$J_{e}=\sigma v\times B$$

where σ is blood conductivity, v is blood flow velocity, and B is magnetic flux density. From Ohm’s law vector equation and current continuity theorem, it can be further deduced that:

(2) ∇⋅(σ∇u)=∇⋅(σv×B)

where u is a scalar potential. From the above equation, it can be seen that the interaction between arterial blood flow velocity and external magnetic field will form an induced potential field within a certain range of human body, which is the magnetoelectric effect of blood flow. At this time, the potential signal caused by blood flow can be obtained through the measuring electrode on the skin surface.

#### 2.1.2. Reciprocity Theorem Integral Equation

Rayleigh–Carson reciprocity theorem is often used to describe the relationship between two independent groups of sources and fields [23,24]. In the previous work [20], we derived the integral Equation (3) based on the reciprocity theorem, which was used to describe the relationship between the surface potential signal u(r), blood flow velocity v(r), and magnetic flux density B(r):

(3) $$u\left(r\right)=\int_{V}J_{r}\left(r\right)\cdot \left(v\left(r\right)\times B\left(r\right)\right)dV$$

where r is the spatial position vector of the arterial blood flow area and dV is the volume of the arterial blood flow area. $J_{r}\left(r\right)$ is the reciprocal current density; its physical meaning is the current density field generated by injecting 1 A DC current at the location of the measuring electrode when there is no external magnetic field. The closer r is to the electrode, the larger $J_{r}\left(r\right)$ is. Therefore, when the position of the measuring electrode is determined, $J_{r}\left(r\right)$ is also determined. Some relevant studies also consider $J_{r}\left(r\right)$ as the contribution ability or weight value of flow velocity at different positions to the surface potential signal [18,25].

According to Equation (3), when the magnetic field and flow velocity are not correlated with time, the surface potential signal can be regarded as a function related to space, and the location of arterial blood flow can be reconstructed by using potential data and an image reconstruction algorithm. However, this imaging method is a typical underdetermined and ill-conditioned problem. The higher resolution depends on more electrodes to obtain more surface potential data, and a large number of operations are required in the inversion process, resulting in the slow imaging speed, which is not conducive to real-time imaging. In order to improve the related problems, an arterial profile scanning imaging method based on a linear gradient magnetic field is proposed in this paper.

### 2.2. Combined Coil for Arterial Profile Imaging

According to Equation (3), a surface potential signal is generated by the mutual coupling of blood flow velocity and the magnetic field, which means that when there is no magnetic field in the artery blood flow area or there is an equal and opposite magnetic field on both sides of the center line of the artery blood flow area, the surface potential signal will greatly attenuate or even disappear. According to this difference, a combined coil that can generate a linear gradient magnetic field with a zero magnetic field region is designed, an alternating magnetic field is applied to drive the zero magnetic field area to scan the measuring body parts, and the location of the artery is located by establishing a time–space relationship. This section mainly introduces the structure of the combined coil and the current excitation mode. The implementation of linear gradient magnetic field and zero magnetic field region scanning will be shown in Section 3.

#### 2.2.1. Combined Coil Structure

**Figure 1:**
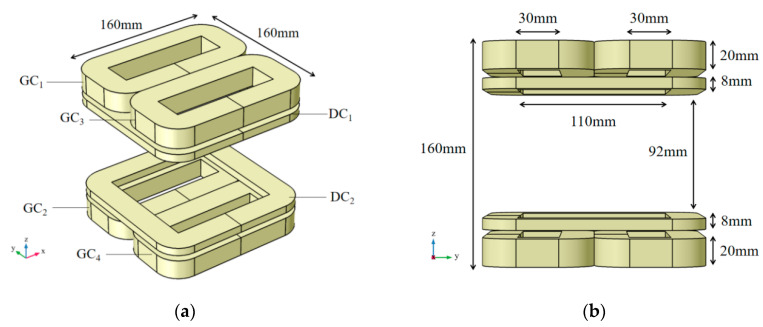
Schematic diagram of combined coil structure. (**a**) 3D view; (**b**) y-z view.

The structure of the combined coil for the arterial profile scanning imaging is shown in Figure 1. It is an open measurement structure, consisting of four gradient coils (GC_1_, GC_2_, GC_3_, and GC_4_) with the same size and two driving coils (DC_1_ and DC_2_) with the same size. The central air area is the measured area, and the measured objects can be entered from the side.

First of all, the same direct current is injected into the coaxial gradient coil, and a direct current of the same magnitude but opposite direction is injected into the gradient coil of different axes, namely $I_{GC_{1}}=I_{GC_{2}}=-I_{GC_{3}}=-I_{GC_{4}}$. The combined coil is established in the Cartesian coordinate system, as shown in Figure 1. The origin of coordinates is located in the center of the air gap area between the upper and lower coils, and the axial direction of the combined coil is in the direction of the *z*-axis. Due to symmetry and superposition of the magnetic field, a linear region with a magnetic field strength of 0 will be generated near the *x*-axis (y = z = 0). The magnetic fields on both sides of the zero magnetic field region have opposite directions and the same intensity. On the whole, it is a linear gradient magnetic field along the *y*-axis. Compared with the circular coil of the same size, the rectangular coil will generate a more uniform zero magnetic field region and a larger linear range, which is conducive to scanning imaging.

In addition, in order to make the zero magnetic field region scan the measured region, two methods can be considered: mechanical control and electrical scanning. The mechanical control method is to drive the gradient coils to move in parallel along the *y*-axis through the mechanical and electrical devices, so that the zero magnetic field region can scan the measured region. However, this method may cause some uncertain errors in the scanning process and slow down the scanning speed. Therefore, the electrical scanning method is adopted in this paper to realize the fast scanning. The realization method is to apply the same low-frequency AC current to the driving coil DC_1_ and DC_2_ at the same time. Then, an alternating magnetic field will be generated in the measured region, and the position of the zero magnetic field region will be changed. The excitation alternating current $I_{drive}$ adopted in this paper is in the following form:

(4) $$I_{drive}=A\cos(2\pi ft)$$

where A is the amplitude of the ac current, which determines the maximum displacement of zero magnetic field region scanning, and f is the alternating frequency. The time required for the zero magnetic field to scan the whole measured region is a half cycle of the alternating magnetic field, that is, the time required for $I_{drive}$ to change from A to −A. Therefore, the relationship between the scanning cycle $T_{S}$ and f is as follows:

(5) $$T_{S}=\frac{1}{2f}$$

Therefore, in the final scanning process, the magnetic field B in the measured region is the superposition of linear gradient magnetic field $B_{G}$ and alternating magnetic field $B_{D}$:

(6) $$B(r,t)=B_{G}\left(r\right)+B_{D}\left(r,t\right)$$

#### 2.2.2. Time–Space Relationship

According to the combined coil and excitation mode in Section 2.2.1, the continuous scanning of the zero magnetic field on the measured region can be realized; thus, a set of continuous time-varying potential signals can be obtained, and the potential signal at each time point can reflect the spatial position of the zero magnetic field. Therefore, the signal acquisition step size $\delta_{TR}$ and the imaging spatial resolution $\delta_{SR}$ have the following time–space relationship:

(7) $$\frac{\delta_{TR}}{T_{S}}=\frac{\delta_{SR}}{W}$$

where W is the total length of the scanning path, and the length scanned by the zero magnetic field within $\delta_{TR}$ is $\delta_{SR}$. Therefore, a set of time–space sequences will be obtained:

(8) $$T=\left[\begin{array}{c} t_{1}\\ t_{2}\\ \vdots \\ t_{N} \end{array}\right];\;Y=\left[\begin{array}{c} y_{1}\\ y_{2}\\ \vdots \\ y_{N} \end{array}\right];\;N=\frac{W}{\delta_{SR}}+1=\frac{T_{S}}{\delta_{TR}}+1$$

where T represents time sequences, Y represents space sequences, and N is the number of sequences. Since the zero magnetic field is scanned along the *y*-axis, the *x*-axis coordinate does not change, so the *y*-axis coordinate $y_{i}$ is used to represent the space position. Thus, when a time point $t_{i}$ is given, we can directly determine its corresponding space position $y_{i}$.

### 2.3. Relationship between Surface Potential Signal and Arterial Radius

**Figure 2:**
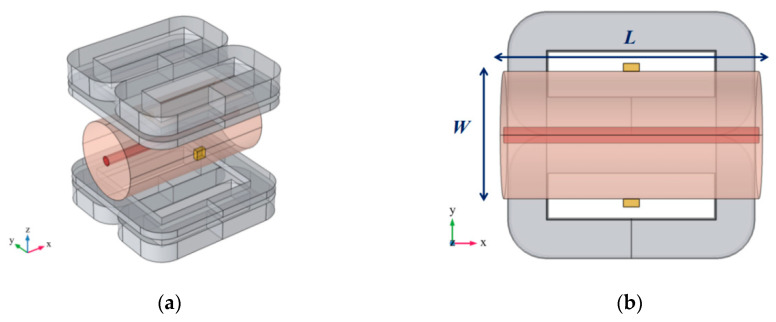
Schematic diagram of surface potential signal simulation measurement in an artery model. (**a**) 3-D view; (**b**) x-y view.

For convenience, the human non-arterial tissue and the artery were respectively equivalent to two cylinders with different radii, as shown in Figure 2, in which the cylinder with a relatively small radius was the area of arterial blood flow, while the cylinder with a relatively large radius was the non-arterial tissue. A pair of measuring electrodes were placed on the surface of the body to detect the surface potential signal. The direction of arterial flow velocity set in the model was the positive direction of the *x*-axis. The magnetic field generated by the combined coil was mainly in the direction of the *z*-axis. The zero magnetic field region in the shape of a line is in the direction of the *x*-axis and the scanning direction is in the direction of the *y*-axis. The profile to be imaged in this study is the x-y plane, as shown in Figure 2b.

According to the magnetoelectric effect of blood flow, the coupling of external magnetic field and arterial blood flow velocity will form an induced electric potential field. At this time, the potential signal can be detected through the measuring electrode on the skin surface. When the external magnetic field is related to time, the generated potential signal is also related to time. Therefore, the reciprocity theorem integral Equation (3) should be extended to the following form:

(9) $$u\left(r,t\right)=\int_{V}J_{r}\left(r\right)\cdot \left(v\left(r\right)\times B\left(r,t\right)\right)dV$$

According to Equation (9), when the magnetic field in the artery blood flow area is zero at $t_{0}$, the potential signal $u\left(r,t_{0}\right)$ is also zero, so the position of the artery center line can be inferred from the moment of zero potential. Since the change of magnetic field is linear and continuous, the generated potential signal is also approximately linear and continuous, which cannot directly reflect the boundary information of the arterial blood flow area. However, in the actual diagnosis of arterial stenosis, it is necessary to observe the radius of the arterial blood flow area. According to Equation (9), it can be preliminarily determined that the amplitude of the potential signal is related to the volume of the integral region. Therefore, the mathematical relationship between the amplitude of the potential signal and the radius of the artery can be considered to solve the problem.

**Figure 3:**
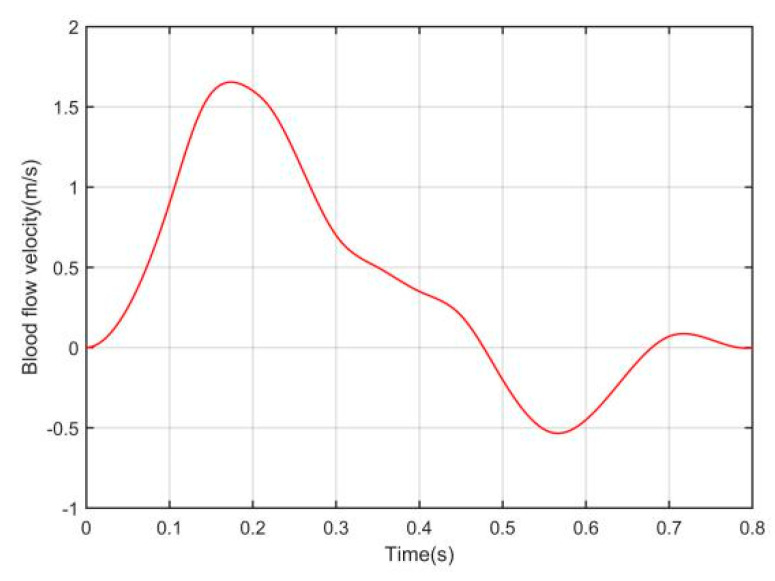
Changes in arterial blood velocity over time during a cardiac cycle [26,27].

In addition, it should be noted that the arterial blood flow velocity v actually changes periodically, but the zero magnetic field scanning cycle $T_{S}$ in this study is far less than the blood flow change cycle. Taking the vertebral artery as an example, the fitting function curve of blood flow velocity v in a cardiac cycle is given in reference [26,27], as shown in Figure 3, where the parameters of the fitting function are determined by a large number of experiments. As can be seen from Figure 3, the cycle time of blood flow change is about 0.8 s, while when the alternating frequency of excitation current is 100 Hz, the scanning cycle $T_{S}$ is only 5 × 10^−3^ s. Therefore, for a study within a scanning cycle, v can be regarded as a constant.

In Figure 2, the direction of the blood flow is along the *x*-axis and the direction of the magnetic field is along the *z*-axis, and they are perpendicular to each other. According to the vector calculation rules, only the y component plays a role in $J_{r}$. Therefore, we can convert the vector parameters in Equation (9) into the form of scalar parameter multiplication:

(10) $$u\left(y_{c},t\right)=\pi r^{2}LJ_{r}\left(y_{c}\right)vB\left(y_{c},t\right)$$

where,

(11) $$J_{r}\left(y_{c}\right)=\frac{1}{L}\int_{-\frac{L}{2}}^{\frac{L}{2}}J_{r}\left(x,y_{c}\right)dx$$

(12) $$B\left(y_{c},t\right)=\frac{1}{L}\int_{-\frac{L}{2}}^{\frac{L}{2}}B\left(x,y_{c},t\right)dx$$

where r is the radius of the artery, L is the length of the measured region as shown in Figure 2, and it is also the length of the zero magnetic field region in the shape of a line. $y_{c}$ is the *y*-axis coordinate corresponding to the center line of the artery. Since the radius of the artery is relatively small, the change of each parameter value in the radial direction of the artery is not significant, so we consider replacing the value of each point in the radial direction with the value of the center line of the artery. $J_{r}\left(y_{c}\right)$ is the average of the y component of the reciprocal current density $J_{r}$ on the line $y=y_{c}$, and $B\left(y_{c},t\right)$ is the average of the z component of the magnetic flux density B on the line $y=y_{c}$. In Figure 2, the origin point of the coordinate system is just at the intersection of the connecting line between two electrodes position and the center line of the artery. Therefore, the integral range of Equations (10) and (11) is from −*L*/2 to *L*/2.

**Figure 4:**
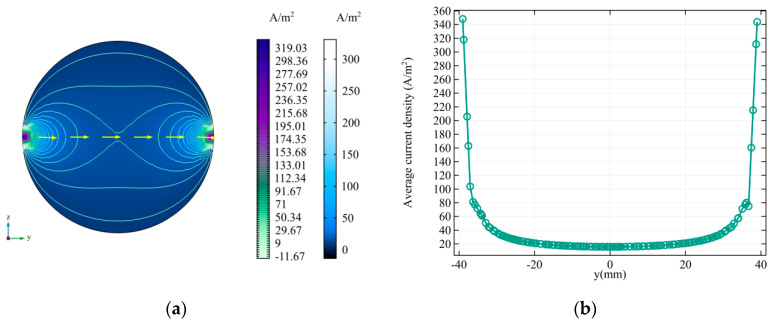
The reciprocal current density distribution on the cross-section. (**a**) y–z views; (**b**) The current density distribution on the connection line of the two electrodes (z = 0).

In fact, $\left[LJ_{r}\left(y_{c}\right)\right]$ in Equation (10) can also be equivalent to the average current density J of a cylinder with a unit axial length. Figure 4 shows the distribution of the equivalent current density J, where the left electrode position is injected with 1 A DC current and the right electrode is grounded. The yellow arrow in Figure 4a is the current flow direction, and the green curve is the isoline of current density. As can be seen from Figure 4b, the values of the reciprocal current density are distributed symmetrically with y = 0 as the center line, the values near the electrode are very large, and the farther from the electrode, the smaller the value. This also indicates that the distance between the arterial blood flow and the electrode is negatively correlated with the contribution of the blood flow to the electrode surface potential signal.

Therefore, Equation (10) can be further simplified as follows:

(13) $$u\left(y_{c},t\right)=\pi r^{2}J\left(y_{c}\right)vB\left(y_{c},t\right)$$

The derivative of the time term in the equation is as follows:

(14) $$\frac{\partial u\left(y_{c},t\right)}{\partial t}=\pi r^{2}J\left(y_{c}\right)v\frac{\partial B\left(y_{c},t\right)}{\partial t}$$

∂u/∂t represents the slope of the time-varying potential signal curve. ∂B/∂t is constant when the magnetic field at $y_{c}$ linearly changes, and v and $J\left(y_{c}\right)$ are also constant. Therefore, the slope of the potential signal curve is mainly related to the radius of the artery. The larger the radius, the higher the slope of the potential signal curve, leading to the greater peak value of the potential signal. According to the excitation current mode in Equation (4), the magnetic field is the largest at the beginning time $t_{s}$ and the end time $t_{e}$ of a scanning cycle, so the amplitude of the potential signal is the largest at the time $t_{s}$ and $t_{e}$. Since the actual position of the artery is not always at the center of the measured region, the potential amplitude at the two moments is not always the same. However, when the scanning cycle has been determined, $\left|u\left(y_{c},t_{s}\right)-u\left(y_{c},t_{e}\right)\right|$ remains unchanged. Therefore, $\left|u\left(y_{c},t_{s}\right)-u\left(y_{c},t_{e}\right)\right|$ and r satisfy the following relation:

(15) $$\left|u\left(y_{c},t_{s}\right)-u\left(y_{c},t_{e}\right)\right|=K_{se}\left(y_{c}\right)r^{2}$$

where the proportionality coefficient $K_{se}$ satisfies:

(16) $$K_{se}\left(y_{c}\right)=\left|K\left(y_{c},t_{s}\right)-K\left(y_{c},t_{e}\right)\right|=\pi J\left(y_{c}\right)v\left|B\left(y_{c},t_{s}\right)-B\left(y_{c},t_{e}\right)\right|$$

In Equation (16), J and B are determined by the structure of the measuring electrode and combined coil structure and the mode of the excitation current, which are called inherent parameters of the system and independent of the measured object. Thus, a linear relationship between the amplitude of surface potential signal and the square of the arterial radius can be established. After the proportional coefficient $K_{se}$ is calculated according to prior knowledge, the radius of the arterial blood flow area can be solved.

### 2.4. Principle of Arterial Profile Scanning Imaging

**Figure 5:**
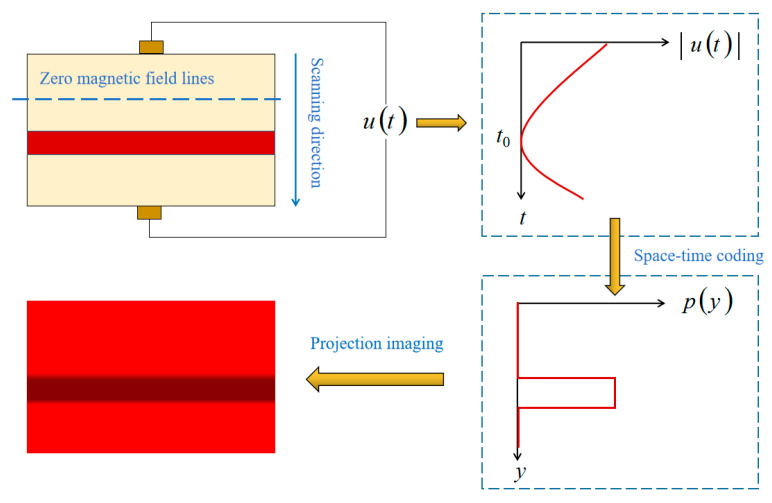
Schematic diagram of arterial profile scanning imaging based on a linear gradient magnetic field.

Arterial profile scanning imaging based on a linear gradient magnetic field mainly includes location imaging and a radius reconstruction of the arterial, and its basic process is shown in Figure 5.

Firstly, the gradient coils are used to generate the linear gradient magnetic field with a zero magnetic field region, and at the same time, the driving coils are used to generate an alternating magnetic field to drive the zero magnetic field region to scan along the *y*-axis, so as to establish the time–space relationship. Then, a time-varying potential signal u(t) is obtained by using measuring electrodes, and the center line position of the arterial is determined by the time characteristic of u(t). Then, the arterial radius is solved by Equation (15), and then the one-dimensional projection sequence p(y) is obtained. Finally, p(y) is projected onto a two-dimensional plane in-line projection to obtain the arterial profile. The implementation steps of transforming potential signals into projection sequences are as follows:

**Step 1:** The surface potential signal sequence is obtained by measurement:

(17) $$U=\left[\begin{array}{c} u_{1}\\ u_{2}\\ \vdots \\ u_{N} \end{array}\right];\;N=\frac{W}{\delta_{SR}}+1=\frac{T_{S}}{\delta_{TR}}+1$$

where $u_{1}$ is the potential value at the first time point of the scanning cycle, and $u_{N}$ is the potential value at the last time point of the scanning cycle.

**Step 2:** Find the time $t_{0}$ corresponding to zero potential, and calculate the scanning distance w of the zero magnetic field region; then, determine the center line coordinate $y_{c}$ of the artery through the time–space relationship (7):

(18) $$w=\frac{\delta_{TR}}{\delta_{SR}}t_{0}$$

(19) $$y_{c}=-\frac{W}{2}+w$$

**Step 3:** Calculate the proportional coefficient $K_{se}$ according to Equation (16).

**Step 4:** Calculate the artery radius r according to Equation (15), and determine the boundary coordinates $y_{b1}$ and $y_{b2}$:

(20) $$r=\sqrt{\frac{\left|u_{1}-u_{N}\right|}{K_{se}\left(y_{c}\right)}}$$

(21) $$\begin{array}{c} y_{b1}=y_{c}-r\\ y_{b2}=y_{c}+r \end{array}$$

**Step 5:** Determine the final projection sequence $P={\left[p_{1}\cdots p_{N}\right]}^{T}$:

(22) $$p_{i}=\left\{ \begin{array}{cc} \left|u_{1}-u_{N}\right| & y_{b1}\leq y_{i}\leq y_{b2}\\ 0 & y_{i}<y_{b1},y_{i}>y_{b2} \end{array}\right.,i=1,\;2\cdots N.$$

## 3. Results

### 3.1. Finite Element Modeling

**Figure 6:**
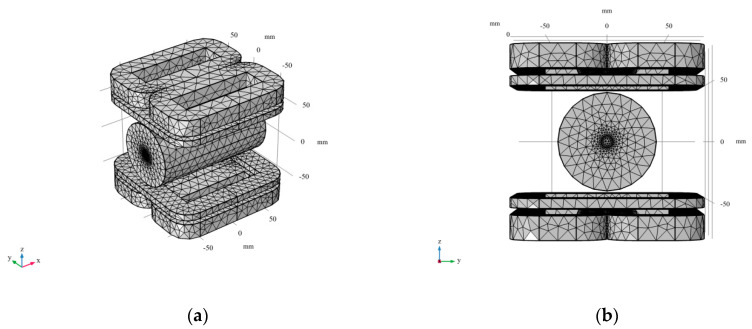
3D finite element simulation model. (**a**) 3D view; (**b**) y-z view.

First, according to the structure of Figure 1 and Figure 2, a 3D finite element simulation model was constructed in COMSOL, including the combined coil model and the single artery equivalent model. The geometric dimensions of the artery model refer to the dimensions of the common carotid artery in reference [27]. The mesh generation structure of the model is shown in Figure 6.

In the study, the arterial model is the area we’re interested in, so we set the arterial model mesh to “Extra fine” in “Predefined” and the combined coil model to “Normal”. The finite element model contains 23,786 mesh vertices and 141,253 tetrahedra. These settings ensure a balance between CPU time and numerical accuracy.

In addition, some necessary parameter settings include the following. The blood flow velocity was set at 0.7 m/s and the electrical conductivity of the artery model was set to 1.09 S/m of the conductivity of the blood (the typical conductivity value) [28]. The material of the combined coil model was set to copper, and its conductivity is 5.998 × 10^7^ S/m. The relative permeability of the whole model was set as 1. The four gradient coils have the same specifications with 500 turns, and the two drive coils have the same specifications with 200 turns. The current applied to the gradient coil group was 10 A, and the amplitude and frequency of the alternating current of the drive coil group were 13 A and 100 Hz, respectively. Therefore, the scanning cycle $T_{S}$ of the zero magnetic field line is 5 × 10^−3^ s, the length W of the scanning path of the zero magnetic field line is 80 mm, and when the expected spatial resolution $\delta_{SR}$ is 1 mm, according to Equation (7), the signal acquisition step size $\delta_{TR}$ should be 6.25 × 10^−5^ s.

### 3.2. Magnetic Field Analysis

#### 3.2.1. Linear Gradient Magnetic Field

**Figure 7:**
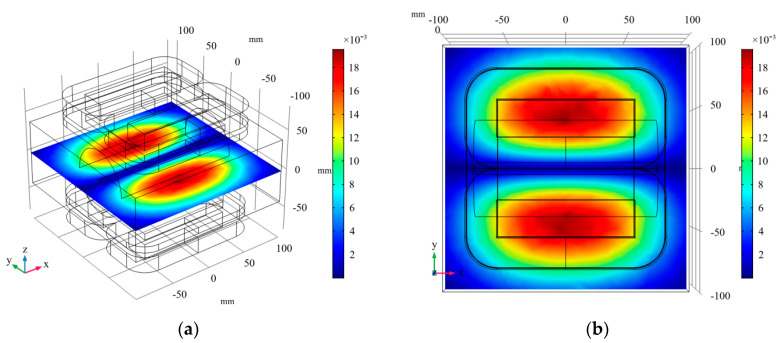
The magnetic flux density modulus distribution of the linear gradient field generated by the gradient coils. (**a**) 3D view; (**b**) x–y view.

When only the gradient coils (GC_1_, GC_2_, GC_3_, and GC_4_) was given 10 A current, the linear gradient magnetic field generated was shown in Figure 7. In order to observe the overall magnetic field distribution in the measured region more clearly, a cuboid air field with a size of 200 mm × 200 mm × 80 mm was added. It can be clearly seen in Figure 7b that the zero magnetic field region in the shape of a line was generated near y = 0, which is caused by the different excitation current directions of coils with different axes. The magnetic flux density modulus on both sides of the zero magnetic field is basically same, but the magnetic field direction is opposite. The x–y plane shown in Figure 7 is the plane to be imaged. When an alternating magnetic field is applied, the zero magnetic field will move along the *y*-axis for scanning.

**Figure 8:**
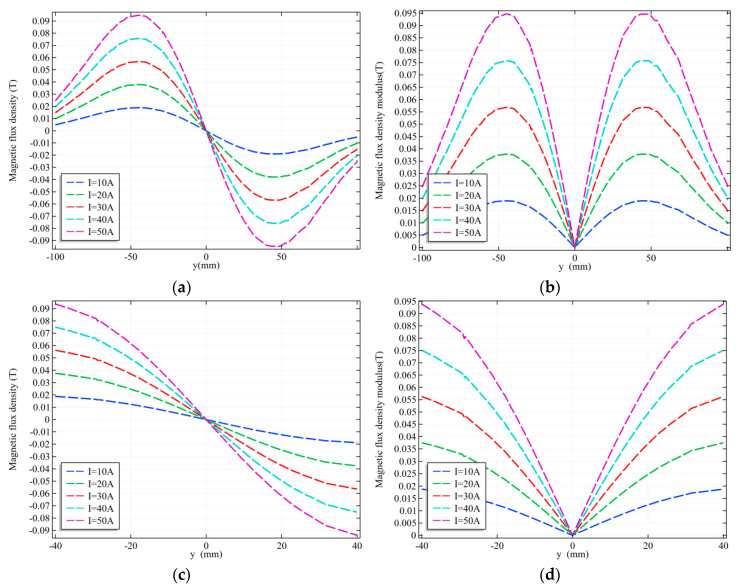
The linear gradient magnetic fields with different excitation currents near x = 0. (**a**) The magnetic flux density of the entire observed region; (**b**) The magnetic flux density modulus of the entire observed region; (**c**) The magnetic flux density within the radial range of the artery model; (**d**) The magnetic flux density modulus values within the radial range of the model.

The relationship between the excitation current $I_{G}$ of the gradient coils and the generated magnetic field $B_{G}$ is shown in Figure 8. Figure 8a,b are the magnetic field distribution on the line between points (0, −100, 0) and (0, 100, 0). Figure 8c,d are the magnetic field distribution on the line between points (0, −40, 0) and (0, 40, 0), namely the artery model range.

As can be seen from Figure 8a, the magnetic field within the scope of action of the combined coil is a sinusoidal wave distribution, reaching its peak value near the central axis of the gradient coil, and the excitation current and magnetic field peak value show a linear relationship. When $I_{G}$ is 10 A, 20 A, 30 A, 40 A, and 50 A, the peak value of the gradient magnetic field $B_{G}$ generated is 0.019 T, 0.038 T, 0.057 T, 0.076 T and 0.095 T, respectively. The linear region between the positive and negative peaks is the linear gradient magnetic field we need, as shown in Figure 8c. The measured object will be placed within this range, and this linearity ensures that the magnetic field at each point in the scanning process of the zero magnetic field can also change linearly with time. Figure 8b,d show good numerical symmetry of the magnetic field on both sides of the zero magnetic field.

#### 3.2.2. Zero Magnetic Field Scanning

**Figure 9:**
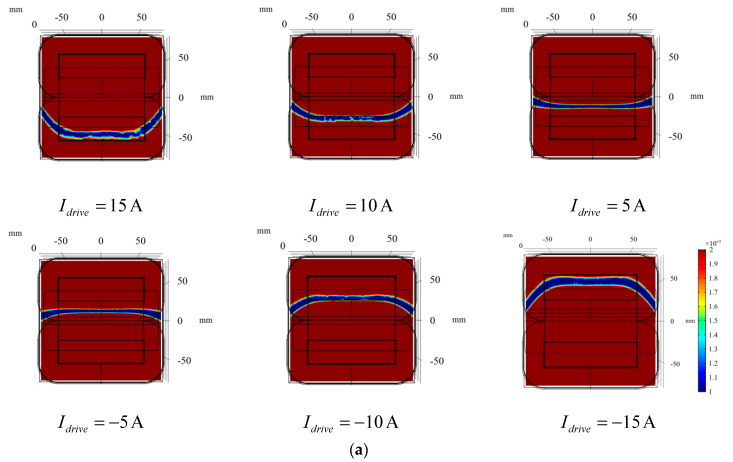

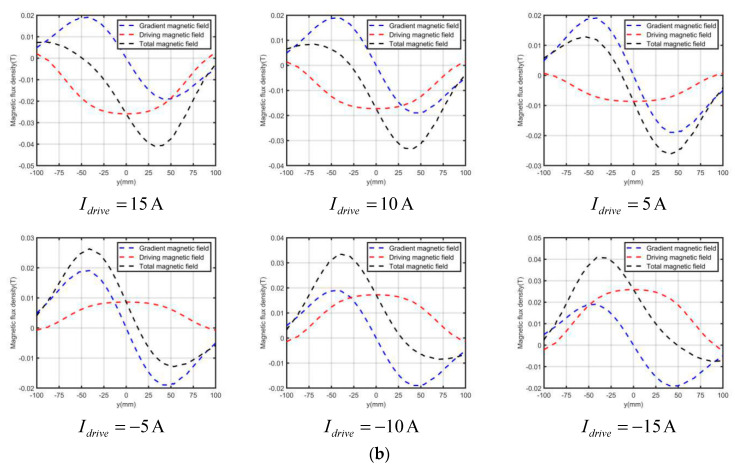
Distribution of magnetic fields with different driving currents. (**a**) position of zero magnetic field region; (**b**) Superposition of linearly gradient magnetic fields with different driving magnetic fields.

After the linear gradient magnetic field and zero magnetic field region are generated from the gradient coil group, an alternating current is injected into the driving coils (DC_1_ and DC_2_) to generate the alternating magnetic field, and zero magnetic field scanning can be realized through the superposition of the magnetic field. The scanning process is shown in Figure 9. Figure 9a shows the positions of the zero magnetic field region when currents with different amplitude are applied to the drive coils, respectively. The color bar is adjusted to highlight the positions of the zero magnetic field region. It can be clearly seen that the zero magnetic field region moves from y = −50 to y = 50 as the excitation current goes from 15 A to −15 A. Figure 9b shows the magnetic field distribution on the *y*-axis corresponding to Figure 9a, showing the details of the superposition of magnetic fields with different driving currents. Therefore, when the cosine excitation method as shown in Equation (4) is adopted for the driving coils, the zero magnetic field region will complete a quick scan on the measured plane within half a cosine cycle. Since the radial range of the artery model was [−40, 40], the excitation current amplitude we chose was 13 A.

**Figure 10:**
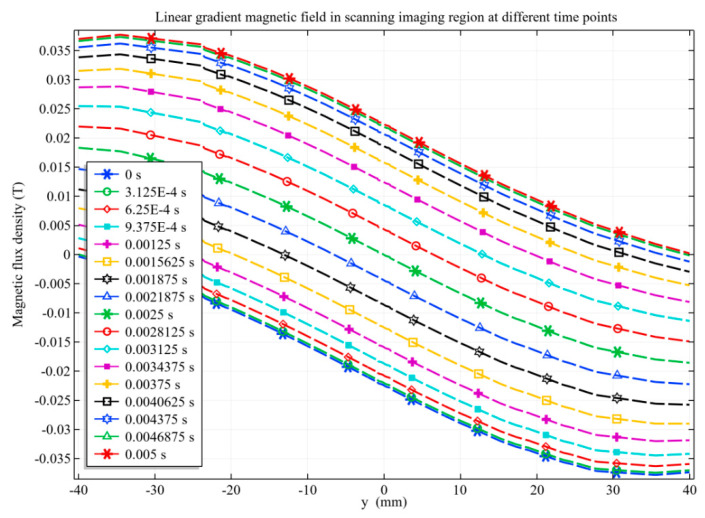
Linear gradient magnetic field distribution at different time points within the range of the artery model.

The magnetic field distribution in the radial direction (along the *y*-axis) of the artery model, namely within the scanning imaging region, is shown in Figure 10. The zero magnetic field moves from y = −40 to y = 40 over time, and the closer to the center, the more uniform the change. The magnetic field of each point changes linearly with time, that is, the rate of change of magnetic field ∂B/∂t is approximately constant.

### 3.3. Scanning Imaging Results

#### 3.3.1. Arterial Position Imaging

**Figure 11:**
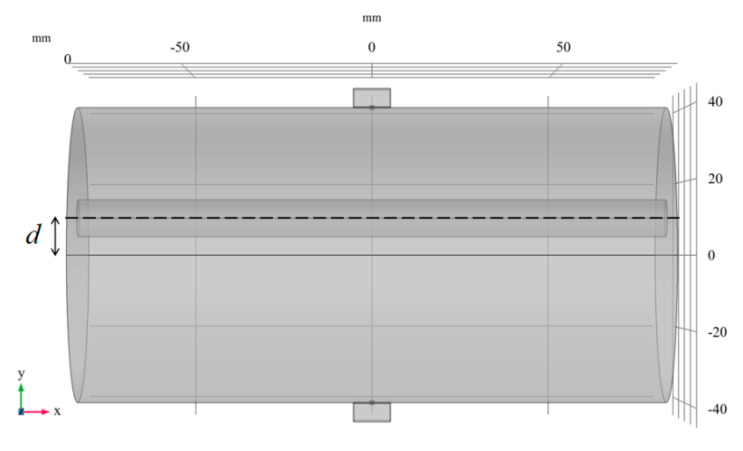
d represents the distance from the center of the artery to y = 0.

**Figure 12:**
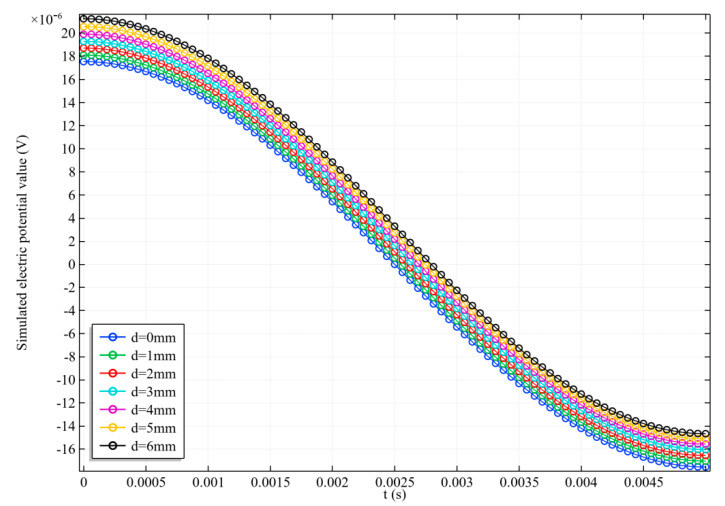
The electrode potential signal during a scanning cycle with different d values.

**Figure 13:**
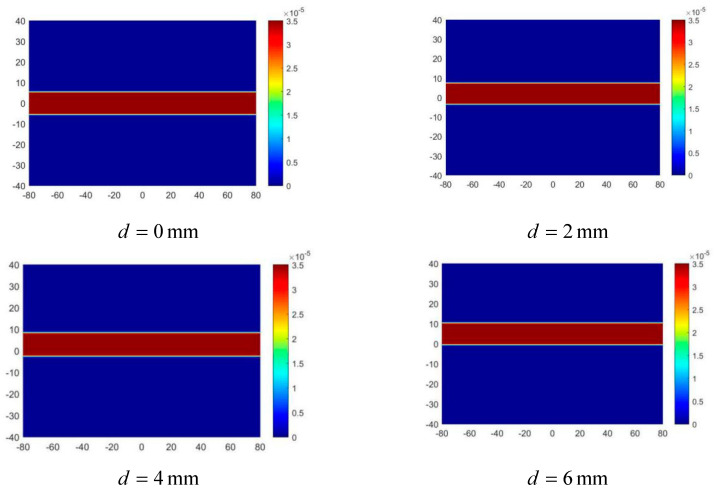
Imaging results of arterial blood flow profile with different d values.

**Figure 14:**
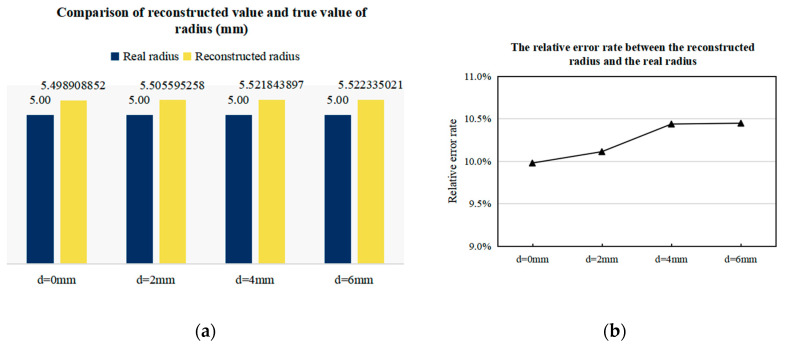
Error of reconstructed value and true value of artery radius with different d values. (**a**) The comparison of the reconstructed radius value with the true value. (**b**) The relative error between the reconstructed radius value and the true value.

As shown in Figure 11, in order to test the sensitivity of the proposed scanning imaging method to the arterial position, different d values were set for simulation, and the radius of the artery was set as 5 mm. The electrode potential signals in a scanning cycle with different d values are shown in Figure 12.

d=0 mm in Figure 12 indicates that the center line of the artery is at y = 0. According to the excitation mode, when t = 0.0025 s, the alternating magnetic field is 0; then, the zero magnetic field is at y = 0, so at this moment, the electrode potential decreases to zero. According to the magnetic field analysis in Section 3.2, the scanning direction of the zero magnetic field is from y = −40 to y = 40. Therefore, when d gradually increases, the artery gradually moves to the positive *y*-axis, and the time corresponding to the zero potential also increases, which is in line with expectations. According to the imaging steps in Section 2.4, the arterial radius r and projection sequence P can be calculated for imaging. The imaging results are shown in Figure 13, and the error analysis between the reconstructed value and the real value of the radius is shown in Figure 14. In order to clearly observe the difference in the position of reconstructed arteries, four images with d values of 0 mm, 2 mm, 4 mm, and 6 mm were presented.

It can be seen from Figure 13 that the reconstructed radius is highly consistent with the true radius, and the reconstruction accuracy is about 90%. Although the reconstruction error increases slightly with the increase of d value, the dispersion degree between the four groups of errors is very low, with the relative error rate between 10% and 10.5%, which indicates that the scanning imaging method proposed in this paper has no specificity for arterial location. In addition, since the absolute error of the four groups of results is around 0.5 mm, it can be speculated that the error may be due to the approximate calculation of $K_{se}$. To verify this hypothesis, arterial models with different r values were set for imaging.

#### 3.3.2. Imaging Results of Arterial Profile with Different Radii

**Figure 15:**
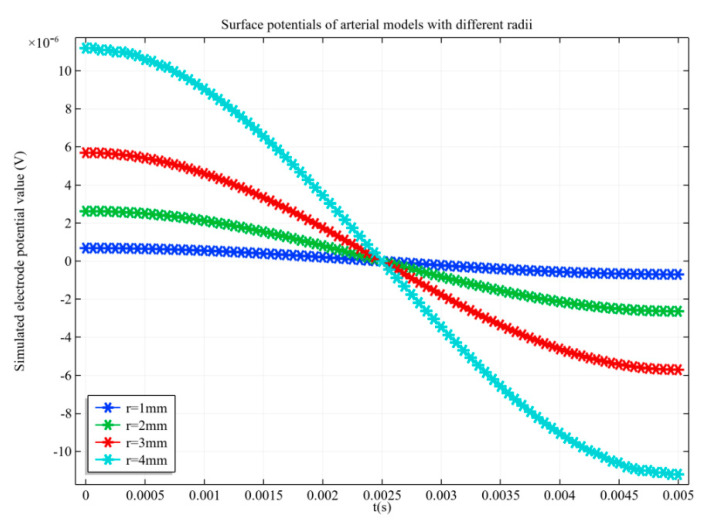
The electrode potential signal during a scanning cycle with different r.

Four groups of artery models with different radius r were set, in which r was 1 mm, 2 mm, 3 mm, and 4 mm respectively, and the center line of the four groups of arteries was y = 0. The electrode potential signal is shown in Figure 15.

**Figure 16:**
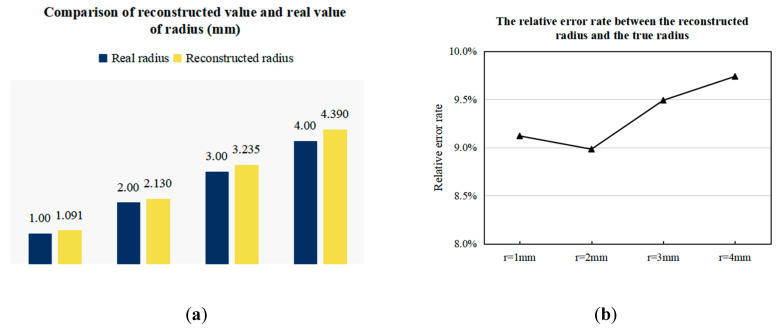
Error of reconstructed value and true value of artery radius with different r values. (**a**) The comparison of the reconstructed radius value with the true value. (**b**) The relative error between the reconstructed radius value and the true value.

**Figure 17:**
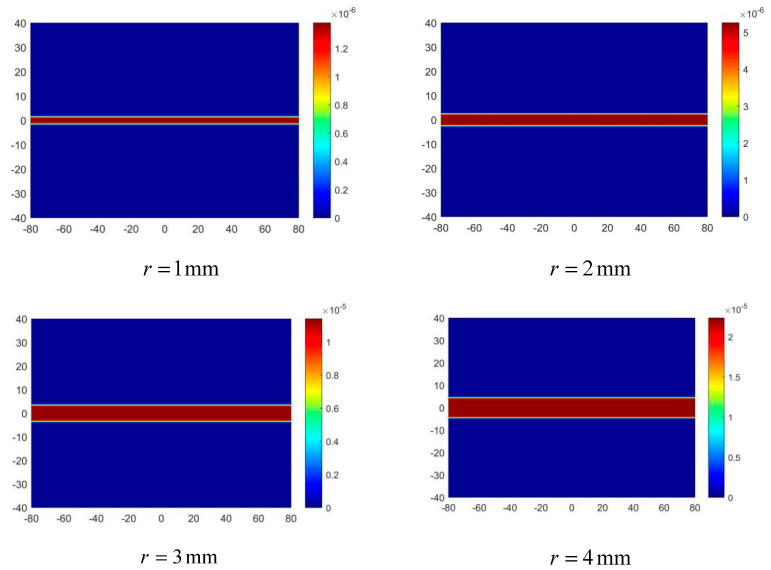
Imaging results of arterial blood flow profile with different r values.

In Figure 15, the zero potential at half of the scanning cycle indicates that the center line of the artery is at y = 0, and with the decrease of r, the slope and amplitude of the potential signal gradually decrease, which is consistent with the analysis in Equation (14). The comparison between the reconstructed value of radius and the true value is shown in Figure 16, and the imaging results of the arterial profile are shown in Figure 17.

It can be seen from Figure 16 that the reconstruction results of the artery model with different radius all have an error of about 9.5%, which further proves that this is an approximate error generated by calculating the $K_{se}$. This error can be improved by adding a correction factor greater than 1 to $K_{se}$. On the other hand, the approximate error decreases with the decrease of the radius. The reason for this phenomenon is that the value of the artery center line is used to replace the value of each point in the radial direction in the imaging process. Therefore, the smaller the radius, the more accurate the approximation. When r = 1 mm, the error increases slightly, because the zero magnetic field region is not a standard line shape, so when the arterial blood flow area is too narrow, the error will increase. In general, the accuracy of radius reconstruction results of each group of models is above 90%, and the spatial resolution is 1 mm, indicating that the scanning imaging method proposed in this paper has high theoretical feasibility.

## 4. Discussion

In this study, firstly, the magnetoelectric effect of arterial blood flow is analyzed, and the reciprocity theorem integral equation used to describe the relationship between detecting voltage signal and arterial blood flow velocity is introduced. Based on the above theory, a novel arterial scanning imaging approach using a linear gradient magnetic field is proposed. The main content of this study includes three parts: generation and scanning of a linear gradient magnetic field, establishment of the numerical model between the voltage signal and artery radius, and the realization of scanning imaging.

Tobias Knopp et al. earlier studied the generation method of free magnetic field lines along a specific direction, proposed a combined coil structure consisting of two mutually perpendicular Maxwell coil pairs [29], and applied it to magnetic particle imaging (MPI) to improve the magnetic field scanning efficiency [30]. Can Barıs Top et al. studied a combined coil similar to this study, mainly analyzing the scanning trajectories of free magnetic field lines in MPI [22]. Koray Ertan et al. studied the coupling effects among gradient coil arrays and proposed a circuit model and analytical formula for driving mutually coupled gradient coil arrays in magnetic resonance imaging (MRI) [31]. According to previous literature research, the gradient magnetic field has been widely used in MRI and MPI, but there is no report on its application in arterial imaging at present. Compared with MRI and MPI, the combined coil in this study is relatively simple and easy to implement. However, with the deepening of the study, the structure and excitation mode of the combined coil may still need to be continuously optimized in further studies.

Although there have been many studies on electromagnetic blood flow measurement [32,33,34], there are few studies on electromagnetic artery imaging. In this study, a numerical model between voltage signal and artery radius under linear gradient magnetic field scanning is derived based on the integral equation of reciprocity theorem. The numerical model and simulation results indicate that the amplitude of the voltage signal is approximately linear with the square of artery radius, which is similar to the research results of Maythem [18]. The difference is that Maythem’ s research conclusion is based on the static excitation mode of the uniform magnetic field, while this study focuses on the scanning excitation mode of the linear gradient magnetic field, so the numerical model includes not only the space term but also the time term. In other words, this study is a supplement and improvement to the existing knowledge system of electromagnetic artery measurement and imaging.

In previous study [20,21], an artery imaging method with uniform magnetic field excitation was proposed. The voltage signal was detected through a 16-electrode array on the body surface, and then the voltage signal was used to solve the distribution of blood velocity inside the artery, after which the two-dimensional section of the artery was reconstructed. However, the results show that this imaging method requires a lot of iterative operations in order to achieve high reconstruction accuracy, and it is usually difficult to reconstruct an image within a few minutes. In this study, the proposed arterial imaging method based on linear gradient magnetic field scanning excitation adopts scanning projection imaging instead of iterative operation. Therefore, the imaging speed is greatly improved, and the research results indicate that the imaging speed is positively correlated with the amplitude and frequency of the alternating current. In theory, hundreds of images can be obtained within 1 s, showing high application potential for the early diagnosis of arteriosclerosis and monitoring arterial expansion and contraction.

Although the arterial imaging method based on a linear gradient magnetic field shows good application potential, this study still has some limitations. First of all, the finite element simulation model constructed in this study only considers the electromagnetic parameters and flow velocity of blood, without considering the influence of fluid dynamics. In fact, the distribution of blood flow velocity in blood vessels is not completely uniform due to the stress of the blood vessel wall and blood. Meanwhile, due to the complexity of the arterial vascular structure, it may require multiple scans to accurately reconstruct the arterial profile. These factors may influence the imaging results and need to be considered in further studies. In addition, in order to verify the clinical validity of the proposed method, more medical data from real patients are needed.

## 5. Conclusions

Through finite element simulation verification, the combined coil proposed in this study can generate a linear gradient magnetic field with a line-shaped zero magnetic field, and it can drive the zero magnetic field to scan in a specific direction under the excitation of the cosine alternating current. The simulation results show that the scanning speed is positively correlated with the amplitude and frequency of the alternating current. When the low-frequency excitation of 100 Hz is applied, the single scanning cycle is 0.005 s, which is far shorter than the arterial blood flow change cycle, meeting the requirements of real-time imaging. Furthermore, the voltage data obtained from each scan can be directly used for projection imaging, without a large amount of inversion calculation, which shows the potential advantage of the proposed method in the real-time imaging of arteries.

In addition, the numerical models (15) and (16) were established between the voltage signal and the artery radius in this study. Combined with the numerical models and the simulated voltage signals, the artery radius can be calculated, and then the arterial profile image can be reconstructed according to the time-space projection imaging method in Section 2.4. The simulation results of the artery models with different radii show that the slope of the voltage signal is proportional to the square of the artery radius in a scanning period, which is consistent with the numerical models and proves the rationality of the numerical models. The imaging results show that the accuracy of reconstruction radius is about 90% and the spatial resolution is 1 mm. Compared with traditional electromagnetic imaging methods, the proposed method has the advantages of fast imaging speed and high resolution, showing the certain application value in early real-time imaging of arterial disease. However, further studies are necessary to confirm its effectiveness in clinical practice by more medical data and real cases
